# 嵌合抗原受体T细胞治疗B细胞肿瘤过程中神经毒性的发生机制及防治策略

**DOI:** 10.3760/cma.j.issn.0253-2727.2021.09.016

**Published:** 2021-09

**Authors:** 琪 张, 毅 肖

**Affiliations:** 华中科技大学同济医学院附属同济医院血液内科，武汉 430030 Department of Hematology, Tongji Hospital, Tongji Medical College, Huazhong University of Science and Technology, Wuhan 430030, China

嵌合抗原受体T细胞（CAR-T细胞）疗法是近年来进展飞速的一种肿瘤免疫治疗方法，广泛应用于血液恶性肿瘤的治疗。CAR分子包括抗体衍生的单链可变片段（scFv）、细胞外铰链区、跨膜结构域以及细胞内信号传导结构域[1]。与T细胞受体（TCR）修饰的T细胞不同，CAR不需要抗原提呈处理或HLA呈递，具有不受限于主要组织相容性复合物（MHC）的特性。细胞内信号传导域被抗原表达的靶细胞激活后会诱导特异性细胞因子分泌，介导炎症反应[2]。

CAR-T相关性脑病综合征（CRES）是CAR-T治疗后的一种常见且严重的不良反应，通常呈自限性[3]，高达64％的CAR-T细胞临床试验可发生CRES[4]–[13]。对100例成年人恶性肿瘤（主要是B细胞肿瘤）患者的研究表明，48％的病例（20％的严重病例）可发生CRES[14]。除CD19外，针对CD22[15]、BCMA[16]–[18]和其他造血抗原[19]的CAR-T细胞治疗过程中也可发生CRES。大多数发生CRES的患者都有细胞因子释放综合征（CRS）病史，因此CRS可被视为CRES的“始发因子”或辅助因子。CRES可能发生在没有CRS的情况下[20]，但严重CRES在经历过CRS的患者中更常见[11],[14],[21]。CRES通常在CRS症状消退后出现[11],[20]，两者同时出现的频率较低[22]–[23]。早期（36 h内）严重CRS伴IL-6和单核细胞趋化蛋白-1（MCP-1）升高提示CRES发生风险高[24]。与CRS相似，CRES在大多数无永久性神经功能缺损的患者中可逆[4],[8]–[9],[11],[25]–[26]。第一阶段与CRS症状相一致，症状较轻，持续时间较短；第二阶段出现在发热和其他CRS症状消退后，症状较重，持续时间较长[25]。对CRES的识别、管理和预防是CAR-T安全、广泛应用的关键。

一、CRES的发生机制

目前对于CRES的病理生理学机制尚不清楚，比较可能的有以下几种：

1. 血管通透性增高、内皮破坏和胶质细胞损伤：血管内皮不仅对体内稳态条件下全身输送氧气和营养物质至关重要，同时也是免疫反应的积极参与者，参与炎症的发生发展[27]。在CAR-T细胞治疗中，血管内皮细胞直接暴露于血流中的多种刺激因素，血管内皮活化有助于CRES的发展。CRES患者表现为内皮细胞的强烈活化，包括血管炎症反应、凝血障碍和血管通透性增强[26],[28]–[29]，提示内皮细胞活化/功能障碍与严重CRES一致，在CAR-T细胞输注前内皮细胞活化的患者更容易发生CRES[28]。据报道，过继免疫治疗后内皮细胞活化和血脑屏障（BBB）破坏直接导致神经细胞损伤[2]，促进CRES进展[26],[29]–[30]。研究发现由CAR-T细胞产生的TNF-α和活化髓样细胞分泌的IL-1b是诱导内皮细胞活化的主要细胞因子，用阿达木单抗和抗IL-1b抗体阻断TNF-α和IL-1b，并应用FAK抑制剂，可以有效改善CAR-T细胞、肿瘤细胞和髓样细胞诱导的内皮细胞活化，并且阿达木单抗和抗IL-1抗体在预防内皮细胞活化方面具有协同作用[31]。此外，血管内皮素在维持BBB（血脑屏障）完整性中起着重要作用。输注CAR-T后早期（第0天和第1天）的Ang2浓度水平与随后的神经毒性严重程度相关，表明内皮细胞活化发生在CAR-T治疗后早期，先于神经毒性发生[26]。尽管内皮细胞在CRES中的关键作用已经被认识到，但具体机制及可能的治疗策略尚未得到很好的研究。总之，在CAR-T治疗期间，患者可能受益于内皮细胞活化的预防[31]。

小胶质细胞吞噬作用在CRES发病机制中的作用以及小胶质细胞表达CD22对CD22 CAR-T细胞治疗的影响尚待阐明，但在患有脑疟疾的儿童中已发现细胞因子介导的小胶质细胞激活[32]，并发展为弥漫性脑病，伴有BBB破坏和脑水肿，临床特征与CRES相似。而多形性胶质母细胞瘤患者鞘内或肿瘤内输注CAR-T细胞时不会发生CRES[2]。

2. 细胞因子及其细胞来源：CAR-T细胞输注并被靶细胞识别激活后，分泌各种炎症因子如GM-CSF，其可激活髓样细胞并促进促炎细胞因子如IL-6和IL-1的产生和分泌，导致炎症毒性[33],[35]–[37]。在严重CRES患者的CSF中观察到髓样细胞的数量显著增加[2]。CRES的发生与患者血清中存在高水平的炎性蛋白和细胞因子，如GM-CSF、IL-6、IL-1b、CRP等有关[9],[26],[28],[38]。血清中IL-1、IL-6、IL-15、TNF-α、IFN-α水平升高与CRES的严重程度呈正相关[12],[25],[39]–[40]，其上升速度以及峰值浓度可预测CRES的发生[41]。由于IL-6、IL-1等细胞因子并非主要由CAR-T细胞产生，靶向细胞因子的免疫调节剂可能不能完全预防CRES。一些研究报告严重CRES患者CSF中的细胞因子（相对于血液）水平过高[11],[42]。CNS内皮细胞的激活导致BBB通透性增加，可能使血液中的细胞因子进入CSF，高浓度的促炎细胞因子（如IL-6、IFN-γ和TNF-α）[26],[29],[43]可能激活脑血管内皮细胞和BBB，导致其通透性增高和随之而来的脑水肿[41]。CRES患者脑脊液中蛋白、CD4^+^ T细胞、CD8^+^ T细胞和CAR-T细胞水平升高提示BBB的完整性丧失[25]。临床研究表明，CSF中的CAR-T细胞数量和细胞因子水平与CRES的严重程度有关[11],[20],[40],[44]。细胞因子是由外周产生并进入CNS还是由中枢产生仍不确定，但CRES患者内皮细胞活化和BBB破坏的证据增加了外周细胞因子进入CNS的可能性[41]。CAR-T细胞也被证明可以进入CNS，产生局部细胞因子并损伤CNS[40],[45]–[46]，在CRES患者的CSF中发现有更多的CAR-T细胞[40],[46]–[47]。

二、CRES临床表现

CAR-T细胞输注到出现CRES的时间为4～6 d[9],[11],[14],[41],[48]，峰值出现在第7～9天，平均持续时间为5～13 d[11],[14],[25],[41],[48]，大多数病例在3～8周内消退[49]。

CRES的临床表现多种多样，通常表现为中毒性脑病，大多数症状是暂时的、完全可逆的[11],[50]。早期症状包括注意力减退[14],[20]–[21],[26]、书写困难和表达性语言障碍[11],[14]（尤其是命名困难[25],[51]）、头痛[20],[41],[51]、意识障碍（麻木、嗜睡[11],[14],[41]、迟钝）、嗜睡[25],[41]和震颤[20],[44]，震颤往往是加重性的生理震颤，也有姿势或意向性震颤[14]。轻度脑病的其他特征包括对时间和地点的定向障碍、短期记忆障碍、失用症、幻觉和行为障碍（冲动、情绪不稳定）或失语[11],[14],[51]，有轻微和短暂的视觉症状，通常呈偏头痛的特点[14],[21]。严重病例有癫痫发作[43]、运动障碍[11],[25]–[26]、颅内压升高（ICP）、躁动性谵妄[14]、昏迷和脑水肿[7],[52]–[54]。癫痫发作是CRES相对常见的表现，是严重CRES的一个明确特征[11]。此外还发现毛细血管渗漏综合征（体重增加、低血压、低蛋白血症），伴严重血管功能障碍[43]。

三、CRES的危险因素

CRES的发生、严重程度和持续时间可能受患者情况、肿瘤或治疗等因素的影响[2]。有研究确定了CRES严重性的独立预测因子：骨髓疾病负担、是否有任何预先存在的神经系统疾病[26]、CD4^+^ CAR-T细胞和CD8^+^ CAR-T细胞剂量和峰值扩张[43]、环磷酰胺和氟达拉滨淋巴结清扫。其他因素包括患者的年龄[10],[55]–[56]、微生物群落、代谢组学或细胞因子谱等[28],[57]–[58]。参与T细胞内稳态的关键细胞因子IL-15可用于CAR-T细胞增殖，也与严重CRES的发展之间存在关联[44],[53],[59]–[60]。

CRES的发生率与高肿瘤负荷、肿瘤类型及CRS的严重程度呈正相关[12],[39]，肿瘤负荷可以预测毒性，临床研究发现，具有较高的肿瘤负荷或使用较多数量的CAR-T细胞治疗的患者CRES发生率较高，生存率较低[6],[28]，这可能与CAR-T细胞群的扩增和同步激活有关[7],[10],[28],[61]–[63]。基线炎症水平较高的患者（如CRP、铁蛋白、D-二聚体和促炎细胞因子水平）发生CRES的风险也会增加[55]，在输注CAR-T细胞后可能有更大的炎症反应倾向。减少肿瘤负担和基线炎症状态，调整预处理方案可降低CRES的发生率和严重程度[2]。

CAR分子的设计和制造可以显著影响CAR-T细胞的增殖和细胞因子的分布，从而影响CRES的发生率和严重程度[2],[64]。具有CD28共刺激信号结构域的CAR-T细胞在输注后增殖更快[8]–[9]，其数量比具有4-1BB共刺激信号结构域的产品更早达到峰值[2]，高达45％的患者出现严重CRES[6],[9]–[10],[65]，而使用含4-1BB的CAR-T产品患者中仅13％发生严重CRES[4],[66]。在单采后选择CD4^+^和CD8^+^ T细胞可提高CAR-T细胞的疗效，但同时也导致CAR-T细胞的炎症毒性增加[61]。

与靶向不同肿瘤上表达的其他抗原的T细胞治疗相比，CD19靶向治疗后CRES的发生率更高，这可能与CD19肿瘤细胞的抗原表达水平有关[41]，B细胞以外的其他细胞（如血脑屏障细胞）表达CD19分子是引发CRES的根本原因[67]。

四、CRES诊断

目前，CRES的诊断主要依赖于患者的临床表现，CRP、铁蛋白和CRS症状出现更早和更高的峰值可用于CRES的预测[9],[11],[14],[41],[68]。Karschnia等[45]分析了25例CRES患者血清中急性期蛋白水平，结果显示大多数患者CRP和铁蛋白水平升高，CRP在出现神经症状前达到最高水平，铁蛋白水平在出现神经症状后达到峰值。大多数CRES患者的脑部CT和MRI成像正常[3]。CT有助于排除脑出血、梗死和水肿等诊断。脑脊液检查可排除并发感染和CNS淋巴瘤或白血病[3]。

五、CRES分级

1. 常见不良反应事件评价标准（CTCAE）：CTCAE分级标准是应用最为广泛的药物不良反应分级标准[69]。根据JULIET试验的数据，Schuster等[70]提出CTCAE对CRES的分级并非最佳，该标准并不能反映CRES的范围及严重程度，分级主要依赖大量非特异性神经系统和精神事件，且在使用时有高度主观性。

2. CAR-T OX-10和ICE评分：最初的临床脑病评分叫做CAR-T OX-10，包括对患者定向、命名、书写和注意力的评估，而不再根据在住院患者中很难评估的日常生活能力受损来定义脑病的等级，现在已经被简化和修改，称为ICE（免疫效应细胞相关脑病）评分[71]，除测试定向、命名、书写及注意力外，增加了对患者听从指令的评估，例如“伸出2个手指”等简单命令。

3. CAR-T OX CRES：除了CAR-T OX-10，还将视神经乳头水肿、脑脊液开放压力和影像学评估参数纳入CRES分级系统，以检测颅内压升高和脑水肿的迹象。但是该标准在临床应用时不方便实现，尤其是危重患者的ICP测量，且脑脊液开放压力受多种因素影响，可能随时发生变化[72]。

4. ASTCT分级标准：ASTCT的ICANS评估系统是CAR-T OX分级系统的改进版本，该标准将ICE评分、意识水平评估、癫痫发作、运动表现和颅内压升高相结合，将成人与儿童分开管理，更加全面，可行性及客观性更高[73]。

六、管理与干预

对所有CRES患者进行干预的最佳时机尚不清楚，但现阶段研究表明早期干预可能是有益的，早期预防并干预CRS可在一定程度上减少CRES的发生。目前针对CRES的治疗策略侧重于通过使用糖皮质激素或抑制炎症细胞因子（如IL-6或IL-1b）信号通路以减轻整体炎症[74]–[75]，但上述治疗并不能阻止CRES的发生，患者对干预措施的反应也不尽相同[74]。

支持性管理包括禁食和饮水、营养支持治疗和改善1级CRES的神经系统检查（脑电图，每天30 min）[25]。输注CAR-T细胞后，应在血液科病房对患者进行系统监测。建议在CAR-T治疗前和CAR-T细胞输注后的前10天，每天对患者进行神经系统评估[76]。在所有出现神经毒性迹象的患者中，应进行影像学检查，以排除其他诊断和早期脑水肿，并监测病情变化。

1. 糖皮质激素：糖皮质激素可以穿过BBB，通常用于CRES的一线治疗[9]–[10]。常用地塞米松和大剂量甲泼尼龙[77]，剂量和疗程依据CRES等级而定，地塞米松通常用于低级别CRES[76]，重复且大剂量甲泼尼龙主要用于4级CRES[78]，糖皮质激素通常在2～3周内逐渐减量，但应密切监测患者是否复发[77]。

2. IL-6拮抗剂：研究表明，单核细胞和巨噬细胞释放的IL-1和IL-6与CRES有关[33]–[34]。托珠单抗是一种可与IL-6受体（IL-6R）结合人源化单克隆抗体，尽管有证据表明早期出现CRES或并发CRS时联合使用最有益[21],[25]，这可能是与早期BBB通透性增加有关，有助于托珠单抗进入CNS[25]。大多数研究表明，使用托珠单抗似乎不会影响CAR-T细胞的疗效[8]–[10]，它可以治疗CRS，但对CRES几乎没有效果[11]，主要可能是因为托珠单抗是一种大分子单克隆抗体，不能穿过BBB[39],[43],[47],[79]。推测托珠单抗可能增加CSF中IL-6水平，加重神经毒性，而IL-6拮抗剂塞妥昔单抗并不增加CSF中IL-6水平[45],[49],[51]，与托珠单抗不同，它是一种与IL-6直接结合的非克隆抗体，对IL-6有很高的亲和力，能阻止IL-6与受体的结合[80]，在仅存在CRES的情况下可能更有益，因此塞妥昔单抗可作为治疗CRES的首选药物[41]。

3. 抗癫痫发作：关于预防性使用抗癫痫药物的意见仍然存在争议，还没有明确证明其可以减少癫痫并发症[11],[21],[45]。用苯二氮卓和其他抗癫痫药物治疗癫痫紧急发作在大多数情况下似乎有效，但尽管早期服用抗癫痫药物，癫痫发作仍会延长[11],[68]。左乙拉西坦是CRES患者首选抗癫痫药物，其药物相互作用发生率低，心脏毒性风险低，在肝功能不全患者中具有良好的安全性[21],[25]。在治疗时推荐应用糖皮质激素联合左乙拉西坦（500～1000 mg每12 h 1次）治疗。

此外，3级以上CRES患者建议转ICU进一步治疗，必要时考虑机械通气以保护气道，以及通过床头抬高、过度通气和甘露醇/高渗性盐水等[81]治疗来降低脑水肿患者颅内压[72],[82]–[85]。

4. IL-1拮抗剂：Norelli等[33]报道了来源于单核细胞的IL-1和IL-6在小鼠模型CRES的发生和发展中的重要作用。阿那白滞素是一种IL-1R拮抗剂，已被FDA批准用于治疗类风湿性关节炎和其他炎症性疾病[87]。当阿那白滞素与IL-6拮抗剂联合使用时，有助于治疗CRES。虽然使用阿那白滞素治疗CRES需要更多的临床试验，但早期应用阿那白滞素可有效预防CRES[88]。有研究发现通过IL-6R阻断信号通路对CRES无效，但IL-1R拮抗剂可在不影响CAR-T细胞的疗效的前提下消除CRES，可能是由于阿那白滞素能穿过BBB[2]，可见阿那白滞素是一种可以预防CRES，同时保持着潜在抗肿瘤作用的极具发展前景的药物。目前有几个临床试验正在评估阿那白滞素在预防（NCT04359784）和治疗（NCT04148430）中预防CRES的应用[43]。

5. 其他策略：GM-CSF是巨噬细胞和单核细胞激活的细胞因子[20],[78]，可以用单克隆抗体（如仑兹鲁单抗）中和，它可以显著减少CNS中的髓样细胞和T细胞浸润，有助于减轻神经炎症（NI）[89]。它并不影响CAR-T细胞的功能，且通过降低CRS和NI的风险来提高肿瘤活性，CAR-T细胞还可以通过基因工程分泌GM-CSF中和抗体，从而进一步降低神经毒性的风险[90]。另外有研究提出环磷酰胺是糖皮质激素难治性神经毒性的另一种选择[90]。

七、小结

CAR-T细胞治疗在血液系统恶性肿瘤的治疗中取得了巨大的成就，目前已开始应用于实体瘤的研究和治疗。然而不良反应在一定程度上限制了它的临床应用，只有将这些不良反应的发生率和影响降到最低，才能有效地提高CAR-T细胞治疗的安全性。通过有效地预防和治疗这些不良反应，越来越多的肿瘤患者将受益于CAR-T细胞治疗。
